# Observation of Ultranarrow Band Red Photoluminescence from Pure Organic Self‐Assembled T2T Micro‐Rods: A Route to High‐Resolution Light Sources

**DOI:** 10.1002/advs.202518814

**Published:** 2025-11-06

**Authors:** Dayeong Kwon, Sang‐hun Lee, Eunji Lee, Jaejin Hwang, Jaekwang Lee, Jeongyong Kim, Jinsoo Joo

**Affiliations:** ^1^ Department of Physics Korea University Seoul 02841 Republic of Korea; ^2^ Department of Energy Science Sungkyunkwan University Suwon 16419 Republic of Korea; ^3^ Department of Physics Pusan National University Busan 46241 Republic of Korea

**Keywords:** excitons, linewidths, organic semiconductors, photoluminescence, self‐assembly

## Abstract

Ultranarrow emission linewidths and high spectral purity are essential for next‐generation displays and advanced optoelectronic/photonic applications. A red photoluminescence (PL) peak with a full width at half‐maximum (FWHM) of 3.2 nm at 625 nm is reported from pure organic π‐conjugated 2,4,6‐tris(biphenyl‐3‐yl)‐1,3,5‐triazine (T2T) self‐assembled micro‐rods (SAMRs). The sharp PL emission intensifies under prolonged exposure and increased laser power, indicating a photo‐brightening (PB) effect. T2T SAMRs are fabricated via thermal annealing of reprecipitated T2T, which facilitates the molecular‐scale reorganization of T2T molecules into ordered domains, thereby promoting high‐quality π‐conjugated crystalline structures. Structural and spectroscopic analyses—including Raman spectroscopy, grazing‐incidence wide‐angle X‐ray scattering, and density functional theory calculations—reveal that the narrow 625 nm PL originates from self‐trapped excitons (STEs) within an ordered *J*‐aggregated triclinic lattice framework. Additionally, upon PB, a linear increase in STE PL intensity with laser power, along with the prolonged exciton lifetime, is observed for single‐stranded T2T SAMRs, which distinguishes STE generation from lasing or amplified spontaneous emission. Remarkably, the emission wavelength remains stable across different laser excitation wavelengths (375, 405, 532 nm), heteromolecular systems, and various crystal sizes, underscoring the robustness of the STE state. These findings position T2T SAMRs as promising candidates for high‐resolution, high‐color‐purity red‐light sources.

## Graphical abstract

π‐Conjugated T2T SAMRs display an ultranarrow red PL peak (FWHM = 3.2 nm at 625 nm). Crystalline *J*‐aggregated domains facilitate STEs that lead to photo‐brightening effect. During exposure, emission from the irradiation spot becomes sharper and more intensely red. The stable, narrow PL spectra from STEs in T2T SAMRs indicate promising high‐resolution, high‐color‐purity red light sources.

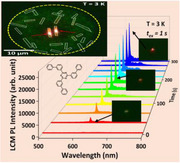

## 1 Introduction

Crafting microscale, high‐resolution light sources with narrow linewidths remains essential for next‐generation augmented reality (AR) and wearable optoelectronic devices, surpassing current LCD, LED, and laser technologies.^[^ ^1^ ^]^ Utilizing self‐emissive materials and amplification techniques is crucial for differentiating them from existing electrically or optically driven gas, liquid, and solid‐state lasers and for enabling full‐angle imaging within a confined viewing range. Unlike traditional lasing, which employs a photonic cavity with mirrors and a gain medium, low‐speckle light‐source materials with high spatial resolution can mitigate coherence‐related limitations inherent to lasers.^[^ ^2^, ^3^ ^]^ Consequently, narrow‐linewidth light sources are well‐suited for advanced AR, holography, flexible optoelectronics, and head‐up‐display systems.

Organic light‐emitting diodes (OLEDs) and π‐conjugated organic semiconductors utilized in displays offer self‐emission, low‐temperature processing, and mechanical flexibility.^[^ ^4^, ^5^, ^6^ ^]^In contrast to inorganic nanomaterials such as quantum dots, organic semiconductors exhibit broader emission linewidths,^[^ ^7^, ^8^, ^9^, ^10^, ^11^ ^]^ which impair color purity and diminish thermal and ambient stability.^[^ ^12^, ^13^ ^]^ These extensive photoluminescence (PL) profiles arise from excitonic interactions with molecular vibrations and variations in molecular packing or solvation.^[^ ^14^, ^15^, ^16^ ^]^ The quantum confinement effect necessary for narrow emission, as observed in inorganic quantum dots, is experimentally unfeasible in organic semiconductors because the effective Bohr radii remain only a few Å owing to the relatively high exciton binding energy (1.5–4.5 eV) and low dielectric constant (2–5) of π‐conjugated organic molecular systems.^[^ ^17^ ^]^ To secure sharp emission, numerous studies have investigated organic solid‐state lasers for their straightforward fabrication and narrow full‐width at half maximum (FWHM). Examples encompass dye‐doped polymer films,^[^ ^7^, ^18^, ^19^ ^]^ organic crystals serving as lasing cavities,^[^ ^20^, ^21^, ^22^, ^23^ ^]^ and external resonators.^[^ ^9^, ^24^, ^25^ ^]^ However, these methods encounter scaling constraints imposed by cavity architectures, and laser coherence leads to speckle interference, which is detrimental to display applications. Therefore, narrow, incoherent PL emission is imperative for microscale light‐emitting devices and photonic systems.

The self‐assembled π‐conjugated organic semiconductors with crystalline order are crucial for developing high‐resolution light‐source materials featuring narrow linewidths. These narrow linewidths result from self‐trapped phenomena of radiative quantum quasiparticles, such as excitons (XF) and excimers (XM). Typically, self‐trapped excitons (STEs) are observed in organic systems exhibiting *J*‐aggregation.^[^ ^14^, ^26^ ^]^ In *J*‐aggregates with significant static disorder, strong electron–phonon coupling induces STEs,^[^ ^11^, ^14^, ^27^, ^28^ ^]^ and the STE PL peak displays a broader linewidth compared to free XF.^[^ ^14^ ^]^ To prevent aggregate‐induced fluorescence quenching and phonon interactions, researchers have introduced spacer groups that inhibit π–π interaction ^[^ ^29^ ^]^ and/or constrained intramolecular motion in conformationally flexible molecules, resulting in aggregation‐induced emission (AIE).^[^ ^8^, ^30^ ^]^ In aggregated systems, the excited‐state transition dipole moments (TDMs) of monomers can couple via Coulomb interactions, delocalizing the excited state across multiple monomers (long‐range TDM coupling).^[^ ^31^, ^32^, ^33^ ^]^ This coupling significantly alters the spectroscopic characteristics of organic aggregates formed by self‐assembled π‐conjugated small molecules, leading to narrowed absorption and fluorescence spectra.^[^ ^34^, ^35^, ^36^ ^]^ For example, Piwonski et al.^[^ ^36^ ^]^ reported absorption and fluorescence linewidths narrowed to 27 and 55 nm, respectively, in the organic compound CzBTCz. Heier et al. reported on the enhancement in room‐temperature photoluminescence quantum yield (PL QY) via morphologically controlled *J*‐aggregates of organic TDBC cyanine dyes in various aqueous environments.^[^ ^10^ ^]^ However, microscale (≈µm) condensed‐state organic light sources with narrow linewidths have not been systematically explored.

In this study, we present organic light‐emitting micro‐rods with high color purity (FWHM = 3.2 nm) and sub‐gap excitation achieved via photo‐brightening (PB) in 2,4,6‐tris(biphenyl‐3‐yl)‐1,3,5‐triazine (T2T) self‐assembled micro‐rods (SAMRs), synthesized by thermal annealing of reprecipitated T2T. Grazing‐incidence wide‐angle X‐ray scattering (GIWAXS) patterns revealed triclinic crystallinity in T2T SAMRs, in contrast to vapor‐deposited thin films. Sharp PL emission at *λ*_em_ = 625 nm with PB was observed under various continuous‐wave (CW) excitations (*λ*_ex_ = 375, 405, 532 nm). Power‐dependent PL, Raman, and time‐resolved PL measurements of individual SAMRs indicated that the 625 nm emission with an ultranarrow linewidth originates from STEs, as supported by density functional theory (DFT) simulations, rather than amplified spontaneous emission (ASE). This emission remained stable for heterogeneous molecular compositions. Therefore, T2T SAMRs hold promise as microscale high‐resolution light sources and advanced flexible photonic/optoelectronic devices.

## 2 Results and Discussion

### 2.1 Structural Characteristics

**Figure 1:**
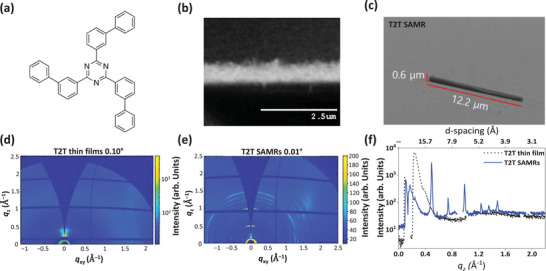
a) Schematic molecule structure of T2T. SEM images of b) vapor deposited T2T thin film and c) T2T self‐assembled micro‐rods (SAMRs). GIWAXS patterns of vapor deposited d) T2T thin films and e) T2T SAMRs. f) Vertical profile of GIWAXS for T2T thin films (black curve) and T2T SAMRs (blue curve).

**Figure** **1**a illustrates a schematic of the T2T molecular structure, and Figure 1b,c shows SEM images of a vapor‐deposited thin film and a T2T SAMR, respectively. During the vapor deposition of T2T thin films, the self‐assembly behavior of T2T molecules produced a rough surface composed of vertically oriented T2T nanorods ≈45 nm in diameter (Figure 1b). Subsequent thermal annealing at 150 °C for 5 min, facilitated the self‐assembly of T2T molecules into SAMRs with 0.58–1.41 µm in width, 0.47–0.73 µm in thickness, and 3.2–13.4 µm in length (Figure 1c). The GIWAXS patterns of the T2T SAMRs revealed crystalline features distinct from those of the deposited T2T thin films, as displayed in Figure 1d,e. Strong scattering signals in the out‐of‐plane direction at *q_z_* = 0.25 Å^−1^, with a shoulder peak at 0.39 Å^−1^, indicated horizontally aligned molecular planes, whereas weak and broad scattering in the in‐plane direction between 1.2 and 2.0 Å^−1^ originated from vertically grown T2T rods. The T2T SAMRs fabricated via thermal annealing exhibited clear diffraction patterns (Figure 1f). Utilizing periodic scattering signals from the GIWAXS measurements, the crystal structures and lattice parameters of the T2T SAMR samples were estimated as described in the Supporting Information (Figures S1 and S2, and Table S1, Supporting Information). For T2T SAMRs, the triclinic crystal structure provided a better fit to the measured lattice spacing (*d*) than the orthorhombic structure (Table S1, Supporting Information).

The triclinic molecular arrangement has been observed in the *J*‐aggregates of ordered or self‐assembled organic molecules with slip‐stacking formation.^[^ ^37^, ^38^, ^39^, ^40^ ^]^ In this study, the characteristic slipped‐stacking arrangement as *J*‐aggregation of self‐assembled T2T molecules was proved by the DFT simulation (Section 2.4), and the GIWAXS measurements of T2T SAMRs confirmed the triclinic crystal structure through the *J*‐aggregation driven by π‐stacking and hydrogen bond.

### 2.2 Photo‐Brightening Effect

**Figure 2:**
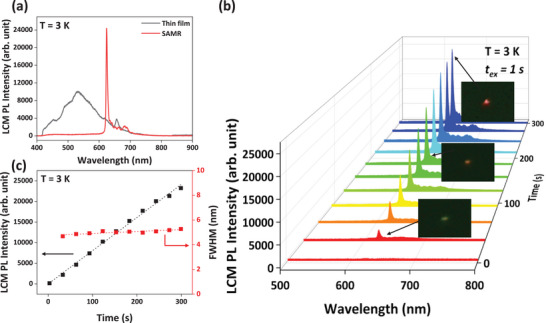
a) LCM PL spectra of T2T thin film (black curve) and a single‐strand T2T SAMR (red curve). b) Time evolution of LCM PL spectrum of T2T SAMR. (Insets: PL emission images at *t* = 30 s (bottom), *t* = 180 s (middle), and *t* = 300 s (top)). The measurement was performed at 3 K using 405 nm CW laser. c) LCM PL intensity and FWHM of a single‐strand T2T SAMR as a function of laser exposure time after the onset of PB effect.

Because the high crystallinity of T2T SAMRs is expected to yield distinctive optical characteristics, the laser confocal microscope (LCM) PL of the deposited thin films and the single‐stranded T2T SAMRs were measured and compared, as shown in **Figure** **2**a. Notably, the LCM PL spectrum (*λ*_ex_ = 405 nm) of a single‐strand T2T SAMR markedly differed from that of a vapor‐deposited thin film at 3 K (Figure 2a). The thin film exhibited broad excimer‐derived PL centered at 530 nm, consistent with existing literature.^[^ ^41^, ^42^ ^]^ By contrast, the SAMR displayed a sharp PL emission at 625 nm (FWHM = 3.2 nm), of which spectral width is similar to lasing or amplified spontaneous emission (ASE).^[^ ^43^, ^44^, ^45^, ^46^ ^]^ Figure 2a shows that the LCM PL peak of the SAMR is both red‐shifted and narrower than that of the thin film, suggesting modifications in molecular packing and/or crystalline structure. The ultranarrow band PL emission intensified with increasing exposure time (*t*) of a 10 µW excitation laser (Figure 2b). Insets in Figure 2b present PL images following the PB effect at *t* = 30, 180, and 300 s for the SAMR. The emission color at the irradiation site shifted to red after 5 min of exposure. These findings indicate that T2T SAMRs exhibit the PB effect under laser irradiation.^[^ ^47^, ^48^ ^]^ Figure 2c illustrates the variations in LCM PL intensity and FWHM as functions of exposure time. After the onset of the PB effect, the LCM PL intensity of a single‐strand T2T SAMR increased linearly under continuous laser exposure, whereas FWHM values remained nearly constant. This indicates a stable, narrow emission with minimal linewidth broadening.

### 2.3 Power‐Dependent PL Characteristics

**Figure 3:**
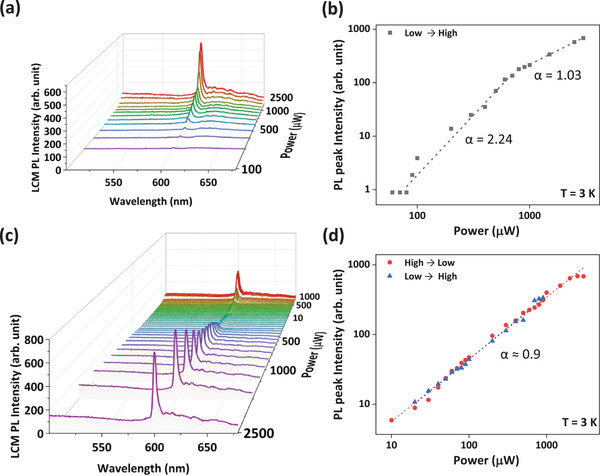
Power‐dependent LCM PL spectra of a single‐strand T2T SAMR: a) Evolution of LCM PL spectra of T2T SAMR with increasing excitation powers for PB effect. b) LCM PL peak intensity versus excitation powers during PB effect at 3 K in a double logarithmic scale. c) Evolution of LCM PL spectra after inducing PB effect. d) LCM PL intensity versus excitation power after inducing PB effect.

To elucidate the origin of the 625 nm PL emission, we conducted systematic measurements of excitation power (*P*_ex_)‐dependent LCM PL spectra on a single‐strand T2T SAMR, as depicted in **Figure** **3**. *P*_ex_ was incrementally varied with high spectral resolution from 10 µW to 3.0 mW under 405 nm excitation. These power increments were selected to capture the threshold behavior and subsequent emission regimes. Below 80 µW, the sharp PL peak at ≈625 nm was absent. Notably, above the 80 µW threshold (Figure 3a,b), PL intensity increased rapidly, signifying the onset of the PB effect. The consistent FWHM throughout the measurements confirms persistent spectral sharpness despite varying excitation. As *P*_ex_ further increased to 800 µW, PL intensity (*I*_PL_) followed a power‐law relationship *I*_PL_ = *I*_0_ *P*_ex_ *^α^* with an exponent *α* = 2.24, indicating PB strengthening. Beyond 800 µW and up to 3.0 mW, *α* stabilized at 1.03 (Figure 3b).

The transition from *α* = 2.24 to near‐unity values reflects a shift from nonlinear to near‐linear PL response under elevated *P*_ex_. Following extensive illumination, the T2T SAMR exhibited a stable PL–*P*_ex_ dependence with *α* ≈0.9 during *P*_ex_ reduction from 3.0 mW back to 10 µW and subsequently demonstrated the same increasing trend upon *P*_ex_ elevation (Figure 3c,d). Throughout the iterative variation of incident laser power, demonstrated an almost linear response (≤ 1.0).

These results of near‐linear response of *I*_PL_ on *P*_ex_ indicate that the sharp PL emission at 625 nm observed after the PB effect was not caused by ASE.^[^ ^49^, ^50^, ^51^ ^]^ Upon triggering the PB effect across laser powers ranging from 10 µW to 3.0 mW, both the FWHM and the 625 nm PL peak position remained nearly unchanged (Figure S3, Supporting Information), highlighting stable emission properties. The enduring 625 nm peak with an ultranarrow spectral width was observed under excitation wavelengths of 375 and 532 nm and across various T2T SAMR sizes (Figures S4 and S5, Supporting Information). The intensity and spectral width of the 625 nm peak under 375 or 532 nm wavelength laser excitations were not as strong and narrow as those observed under 405 nm excitation, most likely due to low laser intensity and photon energy below the T2T bandgap, respectively, indicating that the condition and strength of the PB effect are affected by both the wavelength and intensity of laser illumination. Although optimization and a systematic study of the PB effect in our SAMRs under various laser illumination conditions could be subjects for further in‐depth investigation, the consistent emergence of narrow‐band STE emission across different excitation wavelengths confirms that the emission did not originate from a Raman mode and that the PB effect can be induced by multiple excitation wavelengths. In more than ten distinct T2T SAMR batches, consistent 625 nm PL peaks with ultranarrow linewidths were detected, ruling out defect‐related effects. The peak persisted irrespective of rod dimensions (Section S4, Supporting Information), dismissing contributions from cavity‐based lasing. Temperature‐dependent PL spectra for a single‐strand T2T SAMR (Figure S6, Supporting Information) displayed distinct 625 nm peaks from 3 to 295 K, with the intensity diminishing as the temperature increased (Section S5, Supporting Information).

### 2.4 Density Functional Theory Calculations: *J*‐Aggregation

**Figure 4:**
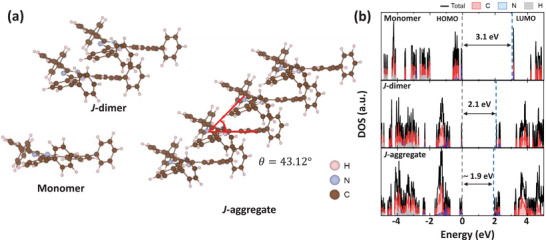
a) DFT‐optimized molecular structures of T2T in the monomer, *J*‐dimer, and *J*‐aggregated configurations. b) Corresponding density of states (DOS) profiles as a function of energy for each configuration.

DFT calculations were conducted to investigate the electronic structures of T2T monomers, dimers, and aggregates (**Figure** **4**a). Prior to electronic analysis, structural optimization identified that the most stable intermolecular arrangement occurs along the *c*‐axis with an intermolecular separation of ≈3.5 Å.^[^ ^52^ ^]^ Utilizing this geometry, we developed a *J*‐dimer model and a representative *J*‐aggregated geometry to analyze the evolution of electronic states upon molecular assembly.

The calculated density of states (DOS, Figure 4b) reveals a progressive reduction in the HOMO–LUMO gap from 3.1 eV in the T2T monomer to 2.1 eV in the *J*‐dimer and 1.9 eV in the *J*‐aggregate. This gap narrowing in the T2T *J*‐aggregate system qualitatively matched the red PL emission of T2T SAMR (Figure 2a). In particular, enhanced orbital overlap within the *J*‐aggregate promotes frontier orbital delocalization leading to LUMO stabilization and a modest upward shift of the HOMO level. The PL peak observed at 625 nm for the single‐strand T2T SAMR closely corresponded to the HOMO–LUMO gap of the *J*‐aggregated system. This agreement between DFT calculation and experimental results suggests that STE within the *J*‐aggregate contribute to the 625 nm emission.

In comparison with previously reported narrow‐band organic emitters, including *J*‐aggregated cyanines and multi‐resonance (MR)‐TADF molecules (FWHM = 9–42 nm at 600–630 nm),^[^ ^10^, ^53^, ^54^ ^]^ the T2T SAMRs exhibited high spectral purity, demonstrating a distinctly narrow emission with a FWHM of 3.2 nm. In previous reports,^[^ ^53^, ^54^ ^]^ PL spectra were measured using a single UV excitation wavelength (320–365 nm), and systematic comparisons under multiple excitation conditions were not conducted.

### 2.5 Time‐Resolved PL and Raman Characteristics

**Figure 5:**
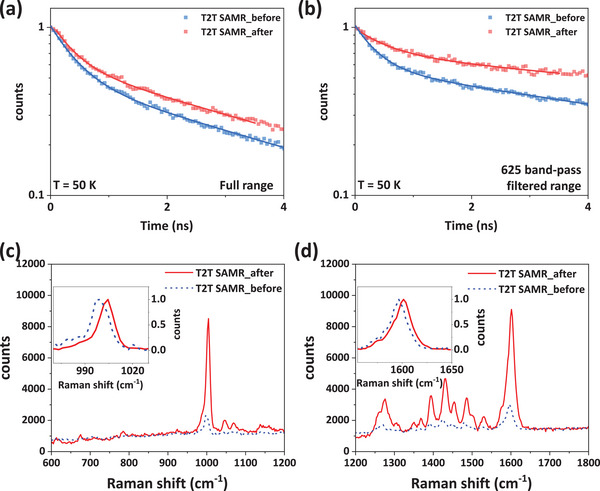
Time‐resolved photoluminescence (Tr‐PL) decay curves for a single‐strand T2T SAMR for a) full range and b) 625 band‐pass filtered range before (blue) and after (red) the PB effect at 50 K vacuum chamber. Raman spectra of T2T SAMRs with (after; red curve) and without (before; blue dotted curve) PB effect, *λ*_ex_ = 532 nm, at 295 K in the range of c) 600–1200 cm^ℒ1^ (Inset: Magnifications of Raman shifts at 999 cm^−1^ corresponding to ring breathing) and d) 1200–1800 cm^−1^ (Inset: Magnification of Raman shift at 1596 cm^−1^ corresponding to ─C═C─ aromatic ring chain vibration).

Time‐resolved photoluminescence (Tr‐PL) decay curves were acquired to investigate the exciton dynamics of the same single‐strand T2T SAMR before and after the PB effect. Tr‐PL decay curves for a single‐strand T2T SAMR were recorded at 50 K using a 375 nm pulsed laser (80 MHz), both with and without a 625 nm band‐pass filter, and were fitted to a bi‐exponential function. Across the full spectral range (no filter), the average exciton lifetimes (*τ*_avg_) were 4.15 ns before PB and 5.04 ns afterward (**Figure** **5**a). With the 625 nm band‐pass filter, *τ*_avg_ increased from 7.43 ns before PB to 11.4 ns after PB (Figure 5b). The prolonged *τ*_avg_ following PB induction indicates a slowed exciton decay. This observation may stem from STE coherence resulting from structural modifications or the activation of additional recombination sites induced by incident laser radiation.^[^ ^55^, ^56^ ^]^

Figure 5c,d display Raman spectra obtained using *λ*_ex_ = 532 nm for T2T SAMRs at 295 K, both before and after the induction of the PB effect. Distinctive peaks were observed around 999, 1268, 1390, 1426, 1446, 1478, 1524, and 1596 cm^−1^, corresponding to ring breathing (999 cm^−1^),^[^ ^57^ ^]^ C─H bending (1268 cm^−1^),^[^ ^58^, ^59^ ^]^ triazine C─N/C═N stretching (1390 cm^−1^),^[^ ^60^ ^]^ C─C ring stretching (1400–1550 cm^−1^),^[^ ^61^ ^]^ and aromatic ─C═C─ chain vibration (1596 cm^−1^).^[^ ^58^ ^]^ Following the induction of the PB effect, additional peaks emerged at 615, 672, 782, 1045, and 1068 cm^−1^, indicative of ring deformation.^[^ ^62^, ^63^, ^64^ ^]^ Concurrently, T2T SAMRs exhibited enhanced peak intensities and reduced FWHM (red curves in Figure 5c,d), signifying increased vibrational coherence and structural ordering.^[^ ^58^, ^59^, ^65^ ^]^ Notably, the ring breathing mode at 999 cm^−1^ narrowed from 11.75 to 10.89 cm^−1^ and experienced a blue shift upon PB induction (inset, Figure 5c), suggesting that the incident laser radiation reinforced ring breathing and intermolecular interactions. Raman and Tr‐PL spectra demonstrated that the molecular packing structure in STE underwent rearrangement, leading to an enhanced PL signal. Additionally, comparing intensity ratios before and after the PB effect revealed that the ─C═C─ aromatic ring chain vibration experienced significant changes (3.0‐fold), whereas other peaks showed increases (≈2.0‐fold) (Figure 5d). This indicates a strong correlation between the ─C═C─ aromatic ring vibrations and the STE peak intensity, potentially because of the resonance effect resulting from molecular rearrangement and/or aggregation.^[^ ^62^, ^66^ ^]^

The mechanism of the laser‐induced PL emission was explained based on the results of incident laser power (*P*_in_)‐dependent LCM PL spectrum, Tr‐PL, and Raman shift. The DFT simulation demonstrated the generation of STEs corresponding to the 625 nm PL through the *J*‐aggregation of self‐assembled T2T molecules with a slipped‐stacking arrangement, supported by the triclinic crystal structure confirmed by GIWAXS measurements.^[^ ^37^, ^38^, ^39^, ^40^ ^]^ The increased exciton lifetime and the enhanced ring breathing mode at 999 cm^−1^ and C═C aromatic ring chain vibration at 1596 cm^−1^ in the Raman spectra indicate strengthened intermolecular interactions and improved local ordering after the PB effect, leading to the generation and coherence of STEs responsible for the ultranarrow red PL emission. A more detailed analysis of the Raman spectra before and after the PB effect is provided in Figure S7 in Section S6 (Supporting Information). Table S2 in the Supporting Information lists the assignments and linewidths of Raman characteristic peaks of T2T SAMRs before and after the PB effect. The defect‐healing effect induced by laser illumination during the PB effect was not clearly observed.

### 2.6 Novel Lighting Material

**Figure 6:**
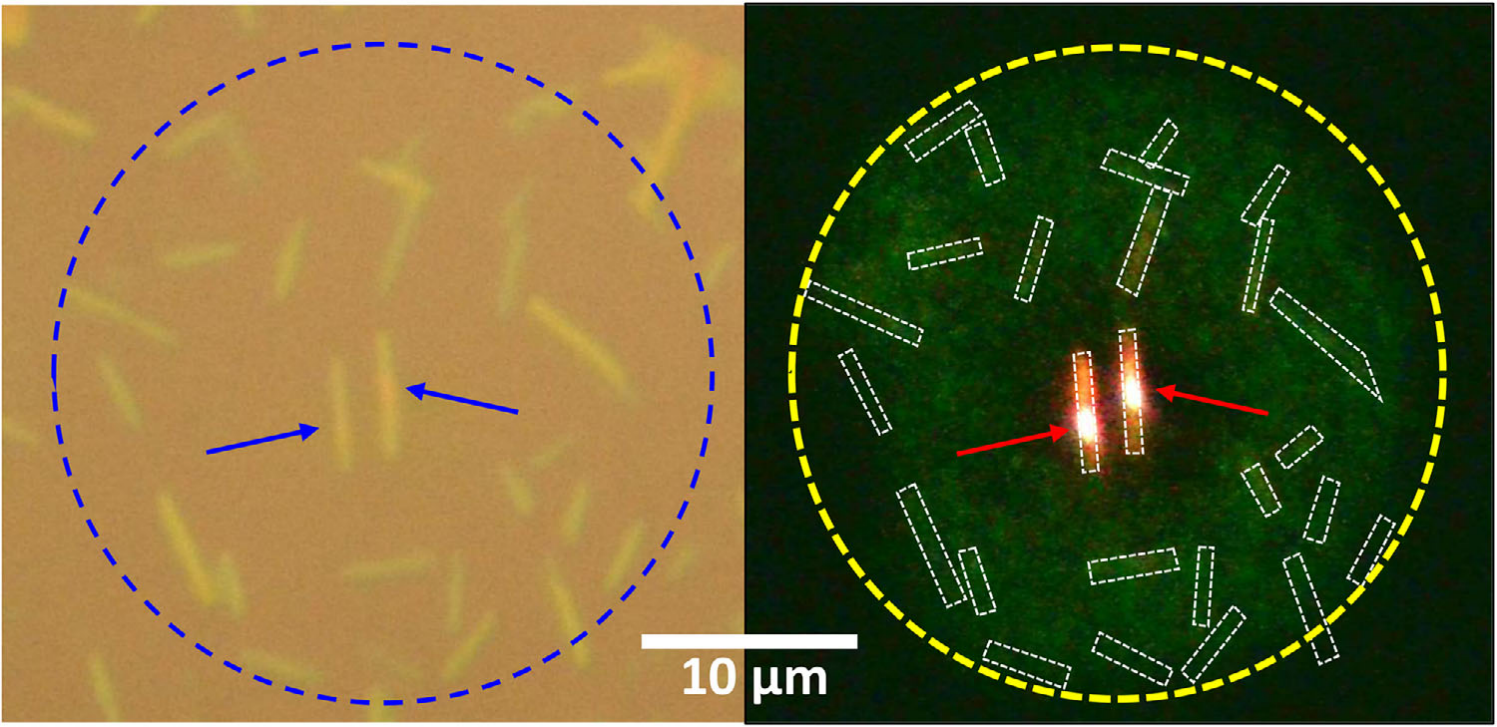
Optical (left) and PL (right) images of T2T SAMRs under 405 nm laser illumination. Selected samples were pre‐irradiated at selected locations on T2T SAMRs (blue arrows) to induce the PB effect and the wider region (yellow dot circle) was illuminated with a lower intensity of 405 nm laser for PL imaging. Only the pre‐exposed T2T SAMRs exhibited strong red emission (red arrows).

The PB effect in T2T SAMRs produced unique emission characteristics, as shown in **Figure** **6**. Bundles of T2T SAMRs were locally irradiated with a focused laser (*λ*_ex_ = 405 nm, *t* = 10 s, *P*_ex_ = 1.0 mW; ≈1 µm spot diameter) to induce the PB effect and modify their emission properties. To directly visualize emission changes, the same laser was illuminated to an ≈15 µm radius (yellow dotted circle), and epi‐PL image were captured from the irradiated area. Only the pre‐exposed T2T SAMRs exhibited bright red emission (arrows in Figure 6), while unexposed SAMRs showed significantly lower PL intensity. This observation suggests that T2T SAMRs could offer a novel approach to high‐resolution light sources.

### 2.7 Comparative Study using m‐MTDATA/T2T Co‐Assembled SAMR

**Figure 7:**
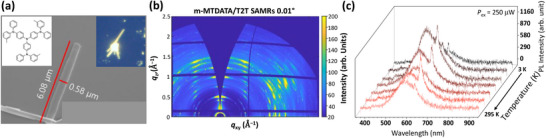
a) SEM image of m‐MTDATA/T2T co‐SAMRs (Insets: Molecular structure of m‐MTDATA (left) and PL image (right)). b) GIWAXS patterns of m‐MTDATA/T2T co‐SAMRs. c) LCM PL spectra of a single‐strand m‐MTDATA/T2T co‐SAMR at various low temperatures.

For comparison, the donor 4,4′,4′′‐tris(N‐3‐methylphenyl‐N‐phenylamino)triphenylamine (m‐MTDATA) was combined with the acceptor T2T to form co‐self‐assembled micro‐rods (co‐SAMRs) through thermal annealing. These co‐SAMRs measured 0.8–1.8 µm in width, 0.5–0.7 µm in thickness, and 6.4–13.6 µm in length (**Figure** **7**a). Bright yellow emission from the m‐MTDATA/T2T junction, owing to the high PL QY of heterojunction exciplex formation,^[^ ^42^, ^67^, ^68^ ^]^ was visible(observed) in the optical microscope image (inset, Figure 7a). Figure 7b shows the GIWAXS pattern of these co‐SAMRs, which displayed sharp diffraction peaks indicative of high crystallinity. Additionally, Figure S8 (Supporting Information) reveals distinct lattice parameters for the m‐MTDATA/T2T co‐SAMRs. Compared to T2T SAMRs, the primitive cell volume decreased from 8.142 nm^3^ (*a* = 25.456 Å, *b* = 20.595 Å, *c* = 15.530 Å; Section S1, Supporting Information) to 3.324 nm^3^ (*a* = 15.43 Å, *b* = 12.89 Å, *c* = 16.71 Å; Section S8, Supporting Information).

To compare the emission characteristics, the LCM PL spectra of a single‐strand m‐MTDATA/T2T co‐SAMR were obtained at various temperatures (Figure 7c). Interestingly, a broad 580 nm peak originated from the charge‐transfer excitons (exciplexes) between m‐MTDATA and T2T.^[^ ^42^ ^]^ Notably, a sharp LCM PL peak at 625 nm was observed for single‐stranded m‐MTDATA/T2T co‐SAMRs (Figure 7c), which was qualitatively consistent with that (originated from STEs) of a single‐strand T2T SAMR. The temperature dependence of the 625 nm PL mirrored that of T2T SAMR.

The LCM‐PL spectra of the m‐MTDATA/T2T co‐SAMRs at various low temperatures revealed simultaneous emissions from donor (m‐MTDATA)–acceptor (T2T) charge transfer excitons (exciplexes; XP) at 580 nm and STEs at 625 nm, as shown in Figure 7c. The emission ratio between XP and STE in the co‐SAMRs showed a noticeable variation with temperature and laser power, while the STE intensity was generally weaker than that of the single‐component T2T SAMRs. This behavior suggests that the presence of donor m‐MTDATA may partially disrupt the *J*‐aggregation of T2T molecules, and that charge transfer leading to XP formation could also reduce the efficiency of STE.

## 3 Conclusion

Purely organic T2T SAMRs were fabricated via reprecipitation and thermal annealing, resulting in ultranarrow band 625 nm PL with the PB effect. The T2T SAMRs display power‐dependent PL, Raman, and time‐resolved PL (Tr‐PL) characteristics that differ from vapor‐deposited films due to the triclinic structures formed via *J*‐aggregation and distinguished exciton species. The linear increase in emission intensity at *λ*_em_ = 625 nm and the stable linewidth with rising excitation power suggest an exciton radiation mechanism distinct from ASE. DFT calculations revealed that the sharp 625 nm PL emission originates from STE through laser‐induced molecular rearrangement, corroborated by Raman and Tr‐PL spectra. Varying excitation wavelengths and PL imaging confirmed the consistent peak position and linewidth of the 625 nm emission, indicating a reliable light‐source material. Spatially localized red PL emission achieved by controlling the optical excitation region demonstrates potential for tunable light sources and photonic device engineering.

## 4 Experimental Section

### Fabrication of Crystalline T2T SAMR

2,4,6‐tris(4‐(pyridin‐4‐yl)phenyl)‐1,3,5‐triazine (T2T, >98.0%) was purchased from Tokyo Chemical Industry and used without further purification. 4,4′,4′′‐tris(N‐3‐methylphenyl‐N‐phenylamino)triphenylamine (m‐MTDATA, >98.0%) and all solvents were procured from Sigma–Aldrich (USA) and used without further purification. The precursor solutions for the T2T SAMRs and m‐MTDATA/T2T co‐SAMRs were prepared using a simple reprecipitation method to induce the aggregation of molecules with a rapid change in solubility. The T2T powders were dissolved in THF (>99.0%) at a concentration of 1 mg mL^−1^ and sonicated (or vortexed) for several minutes to obtain homogeneous precursor solutions for T2T SAMRs. For comparison, precursor solutions for m‐MTDATA/T2T co‐SAMRs were prepared under identical conditions by dissolving premixed powders (1:1 weight ratio) in THF prior to reprecipitation‐induced aggregation. The precursor solutions (1 mL) were rapidly injected into deionized water (10 mL) at 295 K under vigorous stirring (1500 rpm), resulting in an immediate color change from transparent to turbid white. The resulting solutions were deposited onto Si/SiO_2_ substrates (6 × 6 mm, 300 nm SiO_2_ layer thickness) via drop‐casting or spin‐coating (2000 rpm, 20 s) at 295 K and subsequently thermally annealed at 150 °C on a pre‐heated hotplate for 5 min under ambient conditions to produce SAMRs. Alternatively, thermal annealing at 150 °C was conducted in a vacuum oven.

### Measurements

Steady‐state and time‐resolved PL measurements were performed using a custom‐made laser confocal microscope.^[^ ^69^ ^]^ Continuous‐wave excitation lasers (*λ*_ex_ = 375, 405, 532 nm) were focused onto individual T2T SAMR or m‐TDATA/T2T co‐SAMR strands through a 60x objective (NA = 0.7), producing a spot diameter of less than 1 µm. Temperature‐dependent PL measurements were performed in a vacuum chamber equipped with a closed‐cycle cryostat (3–295 K; Montana Instruments Cryostation s50). Spectral dispersion was achieved using a diffraction grating (150 grooves mm^−1^). Raman spectra were collected with a FEX – Confocal Raman Microscopy (NOST) utilizing 532 nm excitations and a 1200 grooves mm^−1^ grating. PL emission images were captured in situ using a TP512000A (ToupTek) digital camera mounted on the microscope. Time‐resolved PL decay traces were measured with a 375 nm pulsed laser (80 MHz, 12.5 ps). Emission signals were filtered through a 390 nm long‐pass filter for the full range and a 625 nm band‐pass filter for the STE region before detection by a Becker & Hickl photomultiplier tube and TCSPC module (Simple Tau).

GIWAXS patterns were obtained at the 3C beamline of the Pohang Accelerator Laboratory using a 1.54 Å X‐ray beam. By maintaining an incident angle of 0.10°, the near‐surface region was investigated. The raw 2D scattering data were analyzed using the beamline software.

### Density Functional Theory Simulation

First‐principles calculations based on density functional theory were performed using the Vienna Ab initio Simulation Package (VASP),^[^ ^70^ ^]^ employing the projector augmented‐wave (PAW)^[^ ^71^ ^]^ within the generalized gradient approximation (GGA) using the Perdew–Burke–Ernzerhof (PBE) functional.^[^ ^72^, ^73^ ^]^ A plane‐wave basis set with an energy cutoff of 450 eV was used, and Brillouin zone integration was carried out using a Γ‐centered 1 × 1 × 1 k‐point mesh, appropriate for molecular systems. Electronic self‐consistency was achieved with a convergence threshold of 10^−6^ eV cell^−1^, and structural optimizations were performed until the residual forces on each atom were below 0.01 eV Å ^−1^. The effect of van der Waals (vdW) interactions was evaluated by applying the Grimme D3 dispersion correction scheme.^[^ ^74^ ^]^ The DFT calculation was performed by using high‐performance computing clusters in the Quantum Matter Core‐Facility (QMCF) of Pusan National University.

## Conflict of Interest

The authors declare no conflict of interest.

## Supporting information



Supporting Information

## Data Availability

The data that support the findings of this study are available from the corresponding author upon reasonable request.
