# Supplementary material for: Observation of Ultranarrow Band Red Photoluminescence from Pure Organic Self‐Assembled T2T Micro‐Rods: A Route to High‐Resolution Light Sources
Source: Adv Sci (Weinh). 2025 Nov 6;13(5):e18814. doi: 10.1002/advs.202518814 (PMC12850107; doi:10.1002/advs.202518814)
Supplement: Supplementary file 1 — Supporting Information [file ADVS-13-e18814-s001.docx]

Supporting Information

Observation of Ultranarrow Band Red Photoluminescence from Pure Organic Self-Assembled T2T Micro-Rods: A Route to High-Resolution Light Sources

Dayeong Kwon, Sang-hun Lee, Eunji Lee, Jaejin Hwang, Jaekwang Lee, Jeongyong Kim*, and Jinsoo Joo*

Section S1. Grazing incidence wide-angle X-ray scattering (GIWAXS) of T2T SAMRs and thin film

In contrast to the GIWAXS pattern of the T2T thin film (Figure 1c), the SAMRs exhibited distinct crystalline reflections (**Figure S1**a). Five vertical line profiles (Figure S1b–f) provided the lattice constants. We converted CCD pixel positions to *d*-spacing values and determined lattice parameters from the peak positions. We obtained triclinic lattice constants *a* = 25.48 Å, *b* = 20.76 Å, *c* = 15.73 Å,^[1]^ adhering to *d*^−2^ = (*h*/*a*)^−2^ + (*k*/*b*)^−2^ + (*l*/*c*)^−2^. The results are presented in **Table S1**. For comparison, we also fitted parameters using an orthorhombic model. The calculated *χ*^2^ value for the orthorhombic structure was 8.6242 × 10^−4^, considerably higher than that of the triclinic structure (3.6191 × 10^−4^), indicating that the triclinic lattice provides a superior fit to the experimental data and *J*-aggregation. GIWAXS profiles of T2T thin films were obtained at various azimuthal angles (**Figure S2**) to evaluate in-plane versus out-of-plane ordering.

**Figure S1.** a) GIWAXS raw data of T2T SAMRs. b–f) Vertical line profiles extracted from *y*-axis pixels 0, 285, 573, 646, and 694, respectively.

**Table S1.** Calculation of *χ*^2^ for *d*-spacing values corresponding to each peak in GIWAXS patterns. (*χ*^2^ calculation was based on triclinic crystalline structure)^[2]^

| *h* | *k* | *l* | *d*_exp_ (Å) | *d*_cal_ (Å) | $\chi^{2}=\left( \frac{d_{cal}-d_{exp}}{d_{cal}} \right)^{2}$ |
| --- | --- | --- | --- | --- | --- |
| 1 | 0 | 0 | 25.607 | 25.478 | 2.5567E-05 |
| 2 | 0 | 0 | 12.718 | 12.739 | 2.7399E-06 |
| 2 | 1 | 1 | 10.818 | 10.817 | 6.2002E-09 |
| 3 | 0 | 0 | 8.4675 | 8.493 | 8.8216E-06 |
| 3 | 0 | 1 | 7.4601 | 7.443 | 5.0743E-06 |
| 1 | 3 | 0 | 6.7073 | 6.660 | 4.9983E-05 |
| 4 | 0 | 0 | 6.3485 | 6.370 | 1.0915E-05 |
| 2 | 3 | 0 | 6.041 | 6.057 | 7.1083E-06 |
| 3 | 1 | 2 | 5.5151 | 5.489 | 2.3100E-05 |
| 0 | 0 | 3 | 5.2815 | 5.240 | 6.3977E-05 |
| 1 | 4 | 0 | 5.0445 | 5.074 | 3.4222E-05 |
| 4 | 0 | 2 | 4.9252 | 4.926 | 5.8218E-08 |
| 2 | 1 | 3 | 4.6347 | 4.666 | 4.6301E-05 |
| 0 | 4 | 2 | 4.2591 | 4.258 | 1.3651E-07 |
| 1 | 3 | 3 | 4.0633 | 4.044 | 2.1694E-05 |
| 2 | 3 | 3 | 3.8899 | 3.893 | 6.2638E-07 |
| 1 | 1 | 4 | 3.7571 | 3.787 | 6.1585E-05 |
|  |  |  |  | *χ*^2^ | 3.6191E-04 |


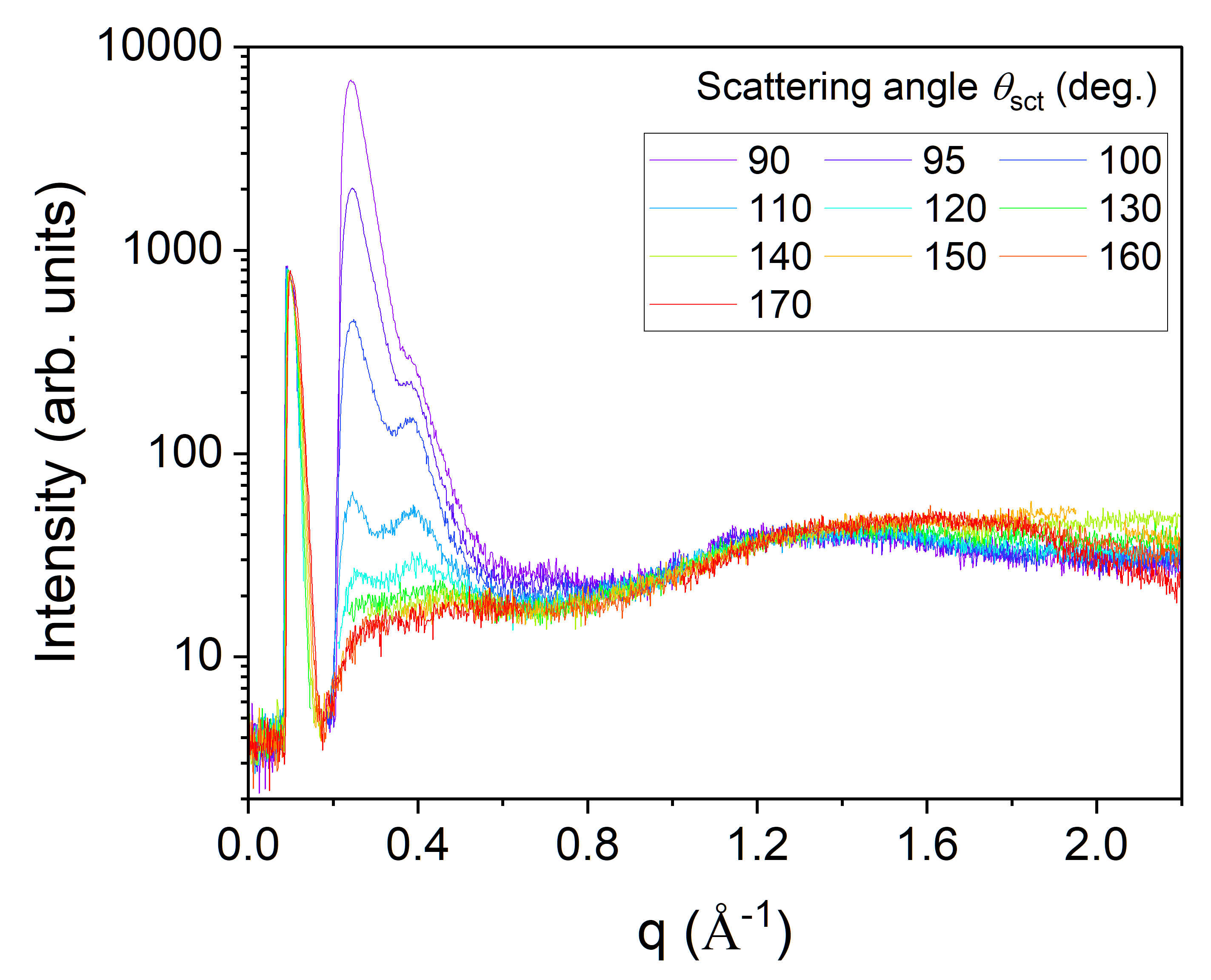


**Figure S2.** Profiles of GIWAXS in various azimuthal angles on detector plane of T2T thin film.

**Section S2. Power dependent PL characteristics: peak position and FWHM**

As presented in the main text (Figure 3), power-dependent PL spectra of a single-strand T2T SAMR were analyzed, with corresponding spectral parameters, including peak position and FWHM, illustrated in **Figure S3**a,b. In line with the PB effect, varying excitation power from 10 *μ*W to 3.0 mW did not alter the peak positions or FWHM, thereby confirming the stability of the 625 nm STE PL emission.

**Figure S3.** For a single-strand T2T SAMR: a) peak positions and b) FWHM of LCM PL as functions of increasing (black markers) and decreasing (red markers) power after the PB effect.

**Section S3. LCM PL spectra of T2T SAMRs under different excitation wavelengths**

Ultrasharp PL at 625 nm was detected for a single-strand T2T SAMR under both 375 nm and 532 nm excitation (**Figure S4**a,b). Notably, with *λ*_ex_ = 375 nm, the emission maintained a FWHM of approximately 4 nm over 10 min (Figure S4a). Additionally, photo-brightening-induced PL enhancement was observed. Under 532 nm excitation, both the emission peak and FWHM remained unchanged, demonstrating that the 625 nm emission is inherently derived from T2T SAMR.

**Figure S4.** LCM PL spectra of a single-strand T2T SAMR under excitation laser a) *λ*_ex_ = 375 nm and b) *λ*_ex_ = 532 nm with various exposure times.

Section S4. LCM PL spectra for different dimensions of T2T SAMRs

Controlling the thermal annealing time altered the dimensions of T2T SAMRs. Annealing at 150 °C for 1 min resulted in rods with a reduced width of 0.429 *μ*m and an extended length of approximately 14 *μ*m compared to those reported in the main text (**Figure S5**a). Increasing the annealing duration to 20 min at 150 °C produced aggregated structures with a width of 2 *μ*m (Figure S5b).

We examined the optical responses of size-modified T2T SAMRs using LCM PL spectra. Figures S5c and S5d show a sharp PL emission at 625 nm from single-stranded T2T SAMRs, regardless of rod dimensions or excitation power. The FWHM remained below 5 nm for all samples. These findings suggest that the ultranarrow 625 nm PL emission arises from the intrinsic properties of self-assembled T2T molecules. Consequently, this emission remains highly stable, indicating its potential as a light source.

**Figure S5.** Optical images of a) relatively thin (width ≅ 429 nm, length ≅ 13.8 *μ*m) T2T SAMRs and b) relatively thick (width ≅ 1.75 *μ*m, length ≅ 20.3 *μ*m) T2T SAMRs. LCM PL spectra of c) relatively thin T2T SAMR and d) relatively thick T2T SAMR (*λ*_ex_ = 405 nm, *P*_ex_ = 1 *μ*W and 1.0 mW).

Section S5. Temperature dependent LCM PL spectra of T2T SAMRs

Sharp PL peaks at 625 nm persisted from 3 K to 295 K (**Figure S6**a). The LCM PL peak position and FWHM increased monotonically with temperature (Figure S6b). The external thermal energy induced molecular vibrations and lattice distortion in organic crystals,^[3,4]^ resulting in temperature-dependent PL in T2T SAMRs. PL emission originating from the STE distinctly emerged at low temperatures and were characterized by an ultranarrow linewidth.

**Figure S6.** a) Temperature dependence of LCM PL spectra of a single-strand T2T SAMR. b) PL peak positions and FWHM of LCM PL spectra as functions of temperature.

**Section S6. Raman peak assignments and linewidths**

**Figure S7**a shows the emergence and/or enhancement of Raman characteristic peaks corresponding to the ring deformation region after the PB process (red curve) in the range of 600 – 830 cm^−1^. Figure S7b presents the normalized Raman spectra before and after PB in the range of 1200 – 1600 cm^−1^, revealing a systematic red-shift of multiple vibrational modes, indicative of enhanced of π–π stacking order. Table S2 lists the Raman peak positions and linewidths.

**Figure S7.** a) Raman spectra of T2T SAMRs with (after; red curve) and without (before; blue dotted curve) PB effect in the range of 600 – 830 cm^−1^. b) Normalized Raman spectra of corresponding sample in the range of 1200 – 1600 cm^−1^.

**Table S2.** Assignments of Raman peaks, linewidth (FWHM) with (before) and without (after) PB effect for T2T SAMRs.^[5–9]^

| Mode assignment | Peak position (cm^−1^) | | Linewidth (cm^−1^) | |
| --- | --- | --- | --- | --- |
|  | Before | After | Before | After |
| Ring deformation | - | 615 | - | 37.9 |
|  |  | 675.2 |  | 7.81 |
|  |  | 786.07 |  | 25.99 |
| Aromatic ring breathing | 999.29 | 1003.40 | 11.75 | 10.89 |
| C–H bending | 1268.10 | 1273.84 | 16.54 | 26.36 |
|  | 1345 | 1351.8 | - | - |
|  | 1363.8 | 1367.3 | - | - |
| Triazine C–N / C=N stretching | 1390.16 | 1392.62 | 14.4 | 16.1 |
| C–C ring stretching | 1424.8 | 1429.8 | 18.1 | 15.8 |
|  | 1450.1 | 1454.7 | 11.8 | 15.4 |
|  | 1478.25 | 1485.55 | 20.9 | 14.5 |
| –C=C– aromatic | 1524.7 | 1530.2 | 22.8 | 17.1 |
|  | 1595.93 | 1600.32 | 23.11 | 21.68 |

**Section S7. Power dependent LCM PL spectra for m-MTDATA/T2T co-SAMRs at various temperatures**

Power-dependent PL spectra were obtained by cyclically varying the incident laser power (*P*_ex_) for a single-strand m-MTDATA/T2T co-SAMR (**Figure S8**). Increasing *P*_ex_ revealed two distinct emission peaks: a broad band around 580 nm, attributed to the exciplex (XP), and an ultranarrow peak at approximately 625 nm, assigned to the STE. As *P*_ex_ increased, the XP emission intensity increased linearly with a stable peak position until the STE PL peak emerged. For *P*_ex_ > 600 *µ*W, XP intensity decreased sublinearly and the peak exhibited a slight red shift. The STE LCM PL intensity demonstrated a superlinear increase with a slope *α* = 3.45 for 600 *µ*W < *P*_ex_ < 1.0 mW, which decreased to *α* = 1.25 when *P*_ex_ exceeded 1.0 mW. The STE PL peak at 625 nm for a single-strand m-MTDATA/T2T co-SAMR remained unchanged across the measured *P*_ex_ range, whereas XP emission showed fluctuating peak positions and linewidths. This behavior suggests that STE spectral stability arises from multiple emissive channels and light-induced exciton population redistribution within co-SAMRs. As *P*_ex_ increased, the STE FWHM at 625 nm narrowed, whereas the FWHM broadened of the XP emission. Additionally, the LCM PL peak position remained constant across the excitation range, highlighting the spectral robustness of the STE state. Linear PL intensity responses and consistent peak positions with narrow FWHMs at 625 nm were observed for STE in m-MTDATA/T2T co-SAMRs, indicating intrinsic STE characteristics in T2T SAMRs.

**Figure S8.** Power dependence of LCM PL spectra of a single-strand m-MTDATA/T2T co-SAMR: a) Evolution of LCM PL spectra of m-MTDATA/T2T co-SAMR with varying excitation powers. LCM PL intensity vs. excitation powers (*P*_ex_) in a double logarithmic scale for b) exciplex (XP, *λ*_em_ = 580 nm) and c) STE (*λ*_em_ = 625 nm).

**Section S8. Grazing incidence wide-angle X-ray scattering (GIWAXS) of m-MTDATA/T2T co-SAMRs**

The m-MTDATA/T2T co-SAMRs exhibited clear crystalline patterns, as shown in **Figure S9**a. Two vertical line profiles were analyzed to determine lattice constants, illustrated in Figures S9b and S9c. Lattice constants were calculated from periodic spacings observed along both profiles, confirming the formation of well-ordered co-SAMR structures. CCD pixel positions were converted to *d*-spacing values, and lattice parameters were estimated from the measured peak positions. The lattice constants are *a* = 15.43 Å, *b* = 12.89 Å, and *c* = 16.71 Å in a triclinic structure, where *d*^−2^ = (*h*/*a*)^2^ + (*k*/*b*)^2^ + (*l*/*c*)^2^. The calculated results are listed in **Table S3**.


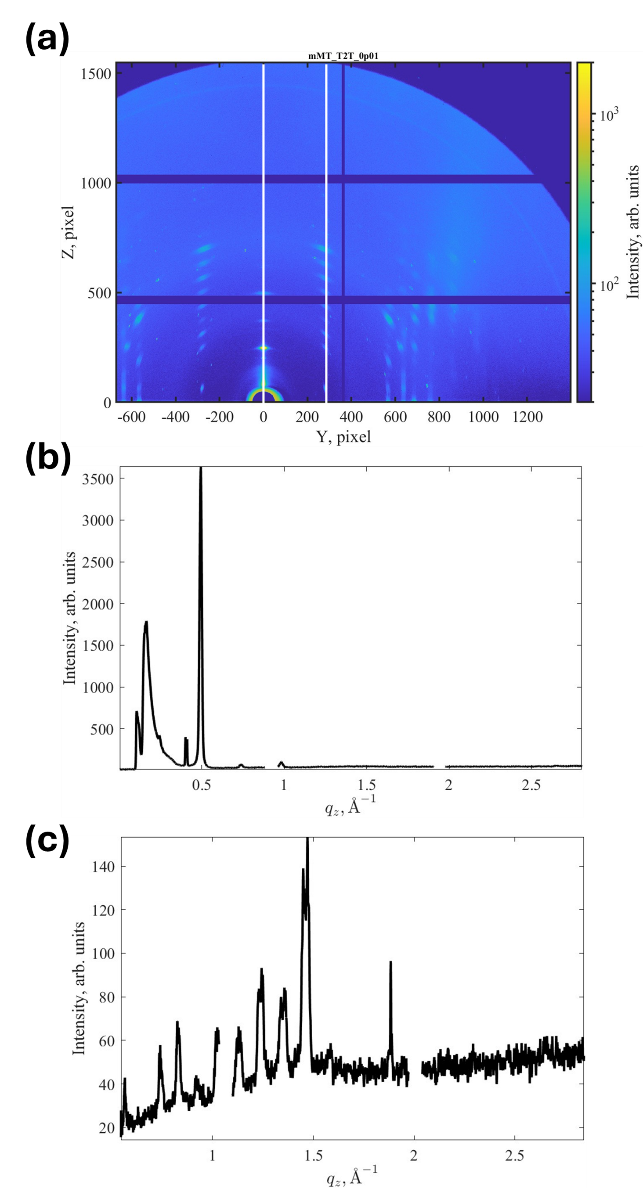


**Figure S9.** a) GIWAXS raw data of m-MTDATA/T2T co-SAMRs. b) and c) Vertical line profiles extracted from *y*-axis pixels 0 and 275, respectively.

**Table S3.** Calculation of *χ*^2^ for *d*-spacing values corresponding to each peak in GIWAXS patterns (*χ*^2^ calculation was based on triclinic crystalline structure).

| *h* | *k* | *l* | *d*_exp_ (Å) | *d*_cal_ (Å) | $\chi^{2}=\left( \frac{d_{cal}-d_{exp}}{d_{cal}} \right)^{2}$ |
| --- | --- | --- | --- | --- | --- |
| 1 | 0 | 0 | 15.49753 | 15.340 | 1.0558E-04 |
| 0 | 1 | 0 | 12.74783 | 12.739 | 4.4477E-07 |
| 1 | 0 | 1 | 11.11151 | 11.106 | 2.6921E-07 |
| 1 | 1 | 1 | 8.51576 | 8.564 | 3.1350E-05 |
| 0 | 0 | 2 | 8.46815 | 8.412 | 4.4321E-05 |
| 2 | 0 | 0 | 7.56919 | 7.670 | 1.7259E-04 |
| 2 | 1 | 0 | 6.80393 | 6.779 | 1.3089E-05 |
| 1 | 1 | 2 | 6.39842 | 6.372 | 1.6815E-05 |
| 2 | 1 | 1 | 6.13629 | 6.201 | 1.0790E-04 |
| 0 | 0 | 3 | 5.56194 | 5.608 | 6.7743E-05 |
| 2 | 2 | 0 | 5.06982 | 5.074 | 7.0087E-07 |
| 3 | 1 | 1 | 4.6547 | 4.619 | 6.1420E-05 |
| 2 | 2 | 2 | 4.29873 | 4.282 | 1.5533E-05 |
|  |  |  |  | *χ*^2^ | 6.3776E-04 |

References

[1] C. Kittel, *Introduction to Solid State Physics*, 8th ed., (Wiley, 2005).

[2] J. L. Devore, *Probability and Statistics for Engineering and the Sciences*, 9th ed., (Cengage, 2015).

[3] A. S. Davydov, *Theory of Molecular Excitons*, (McGraw–Hill, 1971).

[4] M. Pope and C. E. Swenberg, *Electronic Processes in Organic Crystals and Polymers*, 2nd ed., (Oxford University Press, 1999).

[5] P. Løvhaugen, B. S. Ahluwalia, T. R. Huser, and O. G. Hellesø, “Serial Raman spectroscopy of particles trapped on a waveguide,” *Optics Express* 21 (2013): 2964. https://doi.org/10.1364/OE.21.002964

[6] S. G. Jo, D. H. Park, B.-G. Kim, et al., “Dual-mode waveguiding of Raman and luminescence signals in a crystalline organic microplate,” *Journal of Materials Chemistry C* 2 (2014): 6077. https://doi.org/10.1039/C4TC00409D

[7] C.-W. Huang, X. You, P. J. Diemer, et al., “Micro-Raman imaging of isomeric segregation in small-molecule organic semiconductors,” *Communications Chemistry* 2 (2019): 22. https://doi.org/10.1038/s42004-019-0122-7

[8] D. W. Mayo, F. A. Miller, and R. W. Hannah (Eds.), *Course Notes on the Interpretation of Infrared and Raman Spectra*, (Wiley, 2004, pp. 101–140).

[9] X. Chen, Y. Hu, J. Gao, Y. Zhang, and S. Li, “Interaction of Melamine Molecules with Silver Nanoparticles Explored by Surface-Enhanced Raman Scattering and Density Functional Theory Calculations, *Applied Spectroscopy* 67 (2013): 491. https://doi.org/10.1366/12-06838
